# How Do We Motorically Resonate in Aging? A Compensatory Role of Prefrontal Cortex

**DOI:** 10.3389/fnagi.2021.694676

**Published:** 2021-07-29

**Authors:** Sonia Di Tella, Valeria Blasi, Monia Cabinio, Niels Bergsland, Giovanni Buccino, Francesca Baglio

**Affiliations:** ^1^Istituto di Ricovero e Cura a Carattere Scientifico (IRCCS) Fondazione Don Carlo Gnocchi ONLUS, Milan, Italy; ^2^Department of Psychology, Università Cattolica del Sacro Cuore, Milan, Italy; ^3^Department of Neurology, Buffalo Neuroimaging Analysis Center, Jacobs School of Medicine and Biomedical Sciences, University at Buffalo, State University of New York, Buffalo, NY, United States; ^4^Divisione di Neuroscienze, Università Vita e Salute San Raffaele e Istituto di Ricovero e Cura a Carattere Scientifico (IRCCS) San Raffaele, Milan, Italy

**Keywords:** aging, mirror neuron system, magnetic resonance image, rehabilitation, stroke, premotor cortex, prefrontal cortex

## Abstract

Aging is the major risk factor for chronic age-related neurological diseases such as neurodegenerative disorders and neurovascular injuries. Exploiting the multimodal nature of the Mirror Neuron System (MNS), rehabilitative interventions have been proposed based on motor-resonance mechanisms in recent years. Despite the considerable evidence of the MNS’ functionality in young adults, further investigation of the action-observation matching system is required in aging, where well-known structural and functional brain changes occur. Twenty-one healthy young adults (mean age 26.66y) and 19 healthy elderly participants (mean age 71.47y) underwent a single MRI evaluation including a T1-3D high-resolution and functional MRI (fMRI) with mirror task. Morphological and functional BOLD data were derived from MRI images to highlight cortical activations associated with the task; to detect differences between the two groups (Young, Elderly) in the two MRI indexes (BOLD and thickness z-scores) using mixed factorial ANOVA (Group^∗^Index analyses); and to investigate the presence of different cortical lateralization of the BOLD signal in the two groups. In the entire sample, the activation of a bilateral MNS fronto-parietal network was highlighted. The mixed ANOVA (pFDR-corr < 0.05) revealed significant interactions between BOLD signal and cortical thickness in left dorsal premotor cortex, right ventral premotor and prefrontal cortices. A different cortical lateralization of the BOLD signal in frontal lobe activity between groups was also found. Data herein reported suggest that age-related cortical thinning of the MNS is coupled with increased interhemispheric symmetry along with premotor and prefrontal cortex recruitment. These physiological changes of MNS resemble the aging of the motor and cognitive neural systems, suggesting specific but also common aging and compensatory mechanisms.

## Introduction

Mirror neurons are a neural population discovered in the ventral premotor cortex of the monkey (di Pellegrino et al., 1992; Gallese et al., 1996; Rizzolatti et al., 1996a) discharging not only during action execution but also during action observation, configuring a movement observation-execution matching system. Functional magnetic resonance imaging (fMRI) studies provided several evidences for the existence of a Mirror Neuron System (MNS) in humans as well (Hardwick et al., 2018). Specifically, the premotor cortex, particularly the lower part of the precentral gyrus (BA6), the posterior part of the inferior frontal gyrus (BA44), the inferior parietal lobule (BA40), and the superior temporal sulcus (BA22/42) are implicated in human motor resonance and hence can be regarded as part of the human MNS (Rizzolatti et al., 1996b; Grèzes et al., 2001). The MNS is involved in higher motor/cognitive processes, such as the understanding of the meaning of an action and the intentionality of the person executing it, motor learning (Buccino and Riggio, 2006), learning and imitation processes (Jeannerod, 1994; Gallese and Goldman, 1998; Iacoboni et al., 1999; Rizzolatti et al., 2002; Buccino et al., 2004; Vogt et al., 2007; Gallese, 2009), language, and empathy (Antonietti and Corradini, 2013; Oztop et al., 2013; Cook et al., 2014; Rizzolatti and Fogassi, 2014; Buccino et al., 2016). In particular, the MNS operates through a motor resonance mechanism that implies the understanding of the meaning of a gesture through an internal reproduction of the same action in the observer (Fadiga et al., 1995; Strafella and Paus, 2000; Borroni et al., 2005; Borroni and Baldissera, 2008).

Despite the considerable evidence of the MNS’ functionality, further investigation is required to explore its role in aging. Aging is the major non-modifiable risk factor for chronic age-related diseases (Kennedy et al., 2014) associated with mental and physical disabilities for which it is crucial to develop effective rehabilitative interventions. Recently, new rehabilitative interventions for age-related pathologies such as stroke and Parkinson’s disease have been proposed based on the motor resonance mechanism activated by the MNS (Ertelt et al., 2007; Buccino, 2014; Farina et al., 2020). Thus, the comprehension of the mechanisms involved in aging of the MNS is relevant to implement effective rehabilitation programs for age-related pathologies.

Aging is accompanied by structural (for a review see: Vinke et al., 2018) and functional (Cabeza et al., 2002) neural substrate changes and reorganization affecting predominantly the frontal cortex (Raz et al., 1997; Good et al., 2001; Jernigan et al., 2001; Resnick et al., 2003). Functional changes have been observed for both motor and cognitive domains, showing increased activity in contralateral and ipsilateral premotor areas (Calautti et al., 2001; Mattay et al., 2002; Ward and Frackowiak, 2003; Heuninckx et al., 2005, 2008) and bilateralization of activity in prefrontal cortices (Cabeza, 2002). However, relatively little is known regarding physiological changes associated with aging and neurodegeneration within the MNS. Nedelko et al. (2010) reported no age dependent changes in the activity of the MNS, while Farina et al. (2017) found a posterior to anterior shift in activity associated with neurodegenerative decline.

To date however, there is no evidence for a comparison with young people integrating the brain reserve data such as cortical thickness and brain activity within the MNS, defined as the action execution and observation matching system. The purpose of the present study was to investigate these changes in normal aging. We hypothesized that due to the involvement of the MNS in several complex behaviors, ranging from sensory-motor to cognitive and learning processes, the aging of this system would likely involve several brain regions implicated in the aging of both the motor and cognitive systems such as premotor and prefrontal cortices at both the functional and structural level. Specifically, according to literature we expected to find within the MNS of aged subjects cortical thinning, coupled with increased activation and reduced lateralization in the premotor cortices and in the more cognitive-related prefrontal cortices.

## Materials and Methods

### Experimental Design and Statistical Analyses

We employed functional and structural MRI to compare young and elderly participants (between factor) with functional BOLD measures of MNS activity and cortical thickness (within factor). Functional measures for each participant consisted in a conjunction analysis of two conditions, action observation and execution (Price and Friston, 1997; Cabinio et al., 2010; Cerri et al., 2015) to select MNS areas. The functional BOLD signal related to the conjunction analysis derived from regions of interest (ROIs) within the MNS was then extracted and related to the cortical thickness of each ROI. A 2 by 2 ANOVA was used to compare the two groups (between factor) and the two MRI indices, BOLD signal and cortical thickness (within factor).

Sample size was determined based on generally accepted and validated sample size minimums for fMRI studies (Desmond and Glover, 2002) and is in agreement with previous studies from our group (Cabinio et al., 2010).

### Participants

Forty healthy individuals were recruited, consisting of twenty-one young adults [age range 23.20–35.10 years, mean (SD) age 26.66 (3.30) years; 9 females] and 19 elderly participants [age range 57.20–87.60 years, mean (SD) age 71.47 (8.52) years; 11 females]. All participants were preliminarily screened to exclude those with major systemic, psychiatric and neurological illnesses. In elderly participants, conditions associated with cognitive impairment were carefully investigated with the administration of a neuropsychological battery to evaluate general cognitive efficiency: Mini Mental State Examination—MMSE (Measso et al., 1993) (inclusion criteria ≥ 24); language (phonological and semantic fluency) (Novelli et al., 1986); memory (Free and Cued Selective Reminding Test—FCSRT) (Frasson et al., 2011); and attention and executive abilities (Trail Making Test—TMT, part A and B) (Giovagnoli et al., 1996).

All the participants were right-handed as assessed by the Edinburgh inventory (Oldfield, 1971) and were free from psychotropic medications. The study conformed to the ethical principles of the Helsinki Declaration and approved by the Ethics Committee section of “IRCCS Fondazione Don Carlo Gnocchi,” part of the IRCCS Ethics Committee of Regione Lombardia. Informed written consent was obtained from all the included subjects before study initiation. Once included in the study, each participant underwent a single MRI examination, which included structural and functional sequences (see below for details).

### MRI Acquisition

Structural and functional MRI data were acquired during a single session using a 1.5 Tesla Siemens Magnetom Avanto scanner, at Santa Maria Nascente Institute IRCCS, Don Carlo Gnocchi Foundation. Functional images were collected by a gradient echo echo-planar (EPI) T2^∗^ sequence (TR = 3,000 ms; TE = 50 ms; flip angle = 90°; voxel size = 2.8125 × 2.8125 × 4 mm^3^; matrix size = 64 × 64; number of slices = 38; thickness = 4 mm) to detect Blood Oxygenation Level Dependent (BOLD) contrast. Each fMRI session included two runs of 122 volumes. A 3D T1-weighted scan (TR = 1,900 ms; TE = 3.37 ms; voxel size = 1 × 1 × 1 mm^3^; matrix size = 192 × 256; slice thickness = 1 mm; number of slices = 176) was also acquired, to perform volumetric measurements and for anatomical reference in fMRI analysis.

A conventional T2-weighted scan (TR = 2,920 ms; TE = 108 ms; voxel size = 0.75 × 0.75 × 5.2 mm^3^; matrix size = 320 × 320; slice thickness = 4 mm; number of slices = 25) was collected as well, to exclude participants with brain abnormalities. Conventional anatomical sequences (PD-T2, FLAIR) were also executed in order to exclude participants with macroscopic brain lesions and/or more than five white matter hyperintensities (Vale et al., 2015).

### fMRI Experimental Design

**FIGURE 1:**
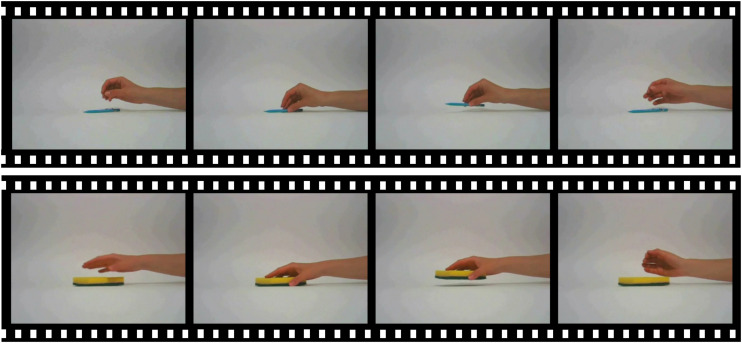
fMRI task stimuli. Sample frames taken from movie clips of a precision grip (upper line): a whole-hand grasp (lower line) used in the observation condition of the fMRI task.

In the course of the fMRI session, subjects were requested to complete 2 block-design runs (i.e., Observation - O- run and Execution - E - run) with an A-B structure (task vs. rest), according to the paradigm described in Cabinio et al. (2010), Farina et al. (2017). In the first run all participants were asked to observe film clips of a right hand executing several grasping movements (O) while in second run participants were asked to execute grasping actions with their right hand according to the object that they viewed on the screen (E). In the O-runs, participants viewed 12 precision-grip movements (e.g., grip a coffee cup from the handle, a key, a pencil) and 12 whole-hand movements (e.g., grasp a torch, a glass, a kitchen sponge). In the E-runs, subjects viewed the pictures of the same 24 objects (see Figure 1). In both the O and the E runs the rest condition was to watch the picture of a right hand at rest. The design was fully randomized (both blocks and runs). Before the fMRI experiment, the participants had a short training session outside the scanner for 15 min. During the training session, participants were also instructed to keep their gaze on the fixation point for the entire duration of the experiment, and to execute grasping actions about once every second. See our previous works for further details (Cabinio et al., 2010; Cerri et al., 2015; Farina et al., 2017). We used an MR-compatible visual system to present the stimuli which included digital goggles (VisuaStim Digital system, Resonance Technology Inc.). The use of E-Prime software (E-Prime 2.0 Psychology Software tool)^1^ ensured exact timing of prompts during MR acquisition. The performance was visually checked by the examiner, who controlled the accuracy and the number of repetitions of the grasping actions during the task execution.

### fMRI Analysis

fMRI data were analyzed according to the General Linear Model with SPM12^2^ running on MATLAB 8.1.0 (MathWork, Natick, MA). Images were first realigned and movement parameters were estimated. Anatomical and functional images were then spatially normalized to the MNI template using a 2 × 2 × 2 mm^3^ voxel size with trilinear interpolation. The normalized functional images were spatially smoothed using an 8-mm full-width at half-maximum isotropic Gaussian kernel. Plots of linear and rotational indices of in-scanner motion were visually inspected to rule out the presence of major artifacts. A threshold of 3 mm or 3 degrees was chosen as limit of acceptable in-scanner motion. Bad volumes were detected and repaired just before estimation using the ArtRepair toolbox^3^. Outlier volumes were first repaired by interpolation to avoid side-effects in the high-pass filtering stage. These volumes were then deweighted in General Linear Model estimation to maintain unbiased estimates.

For first-level statistical analyses, we modeled the expected hemodynamic response function of the software package with a block design. The six parameters related to head movement were included as regressors of no interest. For each participant, we estimated two t-contrasts: observation of a hand grasping (O) and execution of grasping movements (E).

**FIGURE 2:**
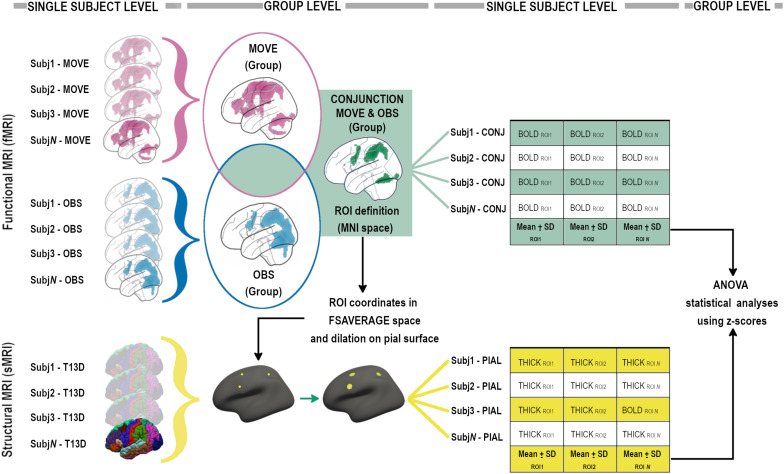
Analysis pipeline, graphical flow-chart representation (see “Materials and Methods” sections for details).

A second level paired *t*-test with both Observation and Execution contrasts was then run. To identify the contour of the MNS, a conjunction (“E” and “O”) analysis, in which the null hypothesis (Nichols et al., 2005) concerns the probability that each voxel is equally activated in both conditions, was performed at the group level using an inclusive mask with both conditions (*p* < 0.001_*u*__*nc*_). To perform group level statistics on the conjunction data, the whole group conjunction activation map was used to define frontal, parietal and temporo-occipital regions of interest (ROIs). Functional ROIs (BOLD-ROIs) were defined as spheres with a 10 mm radius and centered on the peak of activation (see Figure 2).

Conjunction probabilistic maps were then extracted from each participant (single-subject level) considering an inclusive mask with ‘‘O’’ and ‘‘E’’ conditions. The mean signal derived from single-subject level conjunction maps was then calculated for each BOLD-ROI using MARSBAR^4^ (see Figure 2).

The mean signal of each BOLD-ROI was converted into a z-score using the mean and standard deviation of the group and then z-scores were included in statistical ANOVA analyses (see Figure 2).

Lastly, the Juelich Histological Atlas (Eickhoff et al., 2007; Amunts and Mohlberg, 2020), the Harvard-Oxford Cortical Structural Atlas (Desikan et al., 2006), and the Talairach Atlas, non-linearly transformed to convert coordinates from the MNI space to Talairach space^5^ (Lancaster et al., 2007), were used for interpretative purposes.

### T13D MRI Data Analysis—ROIs’ Cortical Thickness

High resolution 3D T1-weighted images were parcellated using the standard recon-all pipeline in FreeSurfer v. 5.3^6^. Quality checks were performed according to ENIGMA guidelines and manual corrections were done when required. Mean thickness was then computed in subject space within selected morphological ROIs (Thick-ROIs), created around the coordinates of the peak of activation of each BOLD-ROI derived from the Conjunction group-level contrast. In order to create circular ROIs in subject space, a multi-step procedure was used (see Figure 2). For each ROI, after conversion of the coordinates from MNI to fsaverage space and the creation of a single-point label, the coordinate was projected on the nearest point on the pial surface and then dilated 10 times forming a circular shape onto the pial surface. Each circular Thick-ROI was then back-projected in subject space and thickness measurements computed creating the Thick-ROIs. Each Thick-ROI measure was converted into a z-score using mean and standard deviation of the group and then z-scores were included in statistical ANOVA analyses (see Figure 2).

### Statistical Analyses

To detect differences between groups for each ROI in the BOLD signal and the cortical thickness, a mixed factorial ANOVA Group^∗^Index was designed. Both Group and Index consisted of 2 levels (Group: Young and Elderly; Index: BOLD z-scores and Thickness z-scores). The statistical analyses were carried out using IBM SPSS version 24. Main effects of Group and Index and interaction effects Group^∗^Index were tested and considered as statistically significant at *p* < 0.05 after applying the Benjamini–Hochberg procedure to control the False Discovery Rate (FDR) (Benjamini and Hochberg, 1995). Simple effects were performed to explore the effect of the independent variable Group within each level of the second independent variable Index.

Moreover, to quantify the degree of lateralization in every subject (see also Cabinio et al., 2010), we used LI-Toolbox software^7^, a toolbox for SPM able to compute the laterality index (LI) (Wilke and Lidzba, 2007). LI was calculated on the basis of the number of voxels surviving the *p* < 0.001 50K threshold, in the right and in the left frontal and parietal lobes separately. LI values ranged between + 1(completely left lateralized) and −1 (completely right lateralized).

## Results

### Sample and Neuropsychological Assessment

Demographics of the study sample are summarized in Table 1. The neuropsychological examination of elderly participants confirmed the absence of cognitive deficits in any explored domain (mean values are above the cut-off scores in all tests as detailed in Table 1).

**TABLE 1:** Demographic data and neuropsychological evaluation of elderly group.

	**Elderly**	**Young**	**Group comparison**	
N	19	21		
Sex (M:F)	8:11	12:9	0.342^a^	
Age (years, mean ± SD)	71.47 ± 8.52	26.66 ± 3.30	<0.001^b^	

**Neuropsychological assessment of Elderly Group**	**Mean**	**SD**	**Minimum–Maximum**	**Cut-off**

MMSE adjusted score (Measso et al., 1993)	27.25	0.99	26.00–29.10	≥23.80
FCSRT—IFR adjusted score (Frasson et al., 2011)	29.30	3.49	22.00–34.40	≥19.60
FCSRT—ITR (Frasson et al., 2011)	35.95	0.23	35.00–36.00	≥35.00
FCSRT—DFR adjusted score (Frasson et al., 2011)	10.09	1.48	7.10–12.00	≥6.32
FCSRT—DTR (Frasson et al., 2011)	11.89	0.46	10.00–12.00	≥11.00
FCSRT—CSI (Frasson et al., 2011)	0.99	0.03	0.85–1.00	≥0.90
Phonemic Fluency adjusted score (Novelli et al., 1986)	38.58	9.11	23.20–57.00	≥17.00
Semantic Fluency adjusted score (Novelli et al., 1986)	41.33	7.80	27.90–58.30	≥25.00
TMT A adjusted score (Giovagnoli et al., 1996)	28.08	19.06	0–66.00	≤93.00
TMT B adjusted score (Giovagnoli et al., 1996)	43.60	41.02	0–145.90	≤282.00
TMT B-A adjusted score (Giovagnoli et al., 1996)	14.97	30.24	0–81.80	≤187.00

*^a^Chi-squared (χ^2^) test.*

*^b^Independent samples Student’s t-test were performed to compare the two groups.*

*MMSE, Mini Mental State Examination; FCSRT, Free and Cued Selective Reminding Test; FCRST-IFR, Immediate Free Recall; FCRST-ITR, Immediate Total Recall; FCRST-DFR, Delayed Free Recall; FCRST-DTR, Delayed Total Recall; FCRST-CSI, Cueing Sensitivity Index; TMT, Trail Making Test; TMT-A, Part A; TMT-B, Part B.*

### Whole Sample fMRI Conjunction Results

**FIGURE 3:**
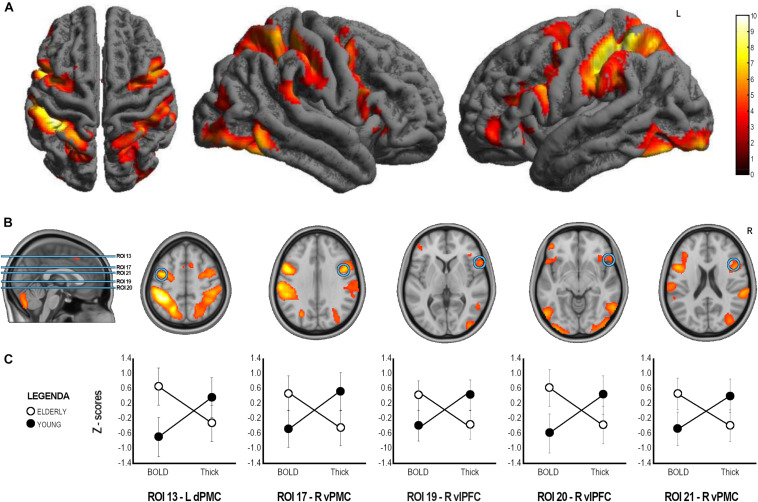
ANOVA analysis results**. (A)** Whole sample fMRI conjunction results (t contrast 3.20; *k* = 50; *p* < 0.001_*u*__*nc*_, see Table 2 for details). **(B)** Brain localization of the ROIs found to have significant Group x Index interaction with significant difference in both cortical thickness and BOLD signal in *post hoc* comparison. **(C)** Graphical representation of BOLD and Thickness Z-scores in the two groups in the statistically significant ROIs. ROI, Region of Interest; L, Left; R, Right; dPMC, dorsal Premotor Cortex; vPFC, ventral premotor cortex; vlPFC, ventrolateral prefrontal cortex.

In the whole sample, to detect the activation of the MNS, fMRI results are explored with the conjunction (action observation and execution) contrast (*t* contrast 3.20; *k* = 50; *p* < 0.001_*u*__*nc*_) showing activation of a bilateral fronto-parietal network formed by the inferior parietal lobule, intraparietal sulcus, postcentral gyrus, middle and inferior frontal gyri; along with activation of the right inferior temporal gyrus, and the bilateral fusiform gyrus. A bilateral cerebellar activation was also detected. All regions of significant activation are summarized in Table 2 and illustrated in Figure 3.

**TABLE 2:**
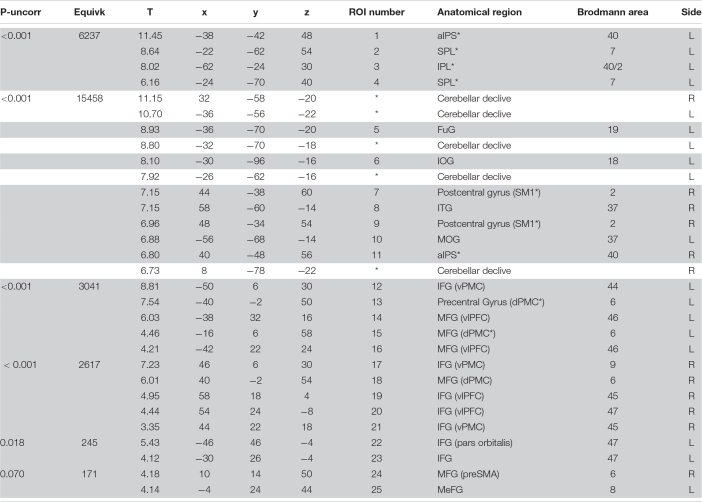
fMRI Conjunction analysis results in the whole sample [threshold *p*_*uncorr*_ < 0.001 with a *k* = 50 contiguous voxels].

In gray colors the coordinates selected as Regions of Interest (ROIs) in subsequent statistical analyses. *Juelich atlas label.

BA, Brodmann area; aIPS, Anterior Intra-parietal Sulcus; SPL, Superior ParietIal Lobule; IPL, Inferior Parietal Lobule; FuG, Fusiform Gyrus; IOG, inferior occipital gyrus; MOG, middle occipital gyrus; SM1, Primary somatosensory cortex; ITG, Inferior temporal gyrus; Middle occipital gyrus; vPMC, Ventral premotor cortex; IFG, Inferior Frontal Gyrus; dPMC, dorsal premotor cortex; MFG, middle frontal gyrus; vlPFC, ventrolateral prefrontal cortex; oPFC, orbital prefrontal cortex; MeFG, medial frontal gyrus; * ROI not included in ANOVA analysis (Group× Index).

Twenty-three ROIs were selected from the conjunction contrast in the whole sample corresponding to the peaks of activation clusters and within each cluster to different cortical gyri or Brodmann areas (BAs) (see Table 3). After that we extracted the mean BOLD signal from single subject conjunction activation maps in the BOLD-ROIs and the cortical thickness from the Thick-ROIs.

**TABLE 3:** Results of the ANOVA Group (Young, Elderly) *Index (BOLD, Thickness z-scores).

							**Group**	**Index**	Group * Index	**Young vs. Elderly**	
**ROI n.**		**Young**		**Elderly**			**F (1,37)**	**F (1,37)**	**F (1,37)**	**Thickness**	**BOLD**
	**Region**	**Z-scores**	**Mean**	**SD**	**Mean**	**SD**	**pFDR**	**pFDR**	**pFDR**	***p*-value**	***p*-value**
ROI 1	aIPS	Thickness	0.27	0.90	–0.28	1.04	2.662	0.000	0.615	0.086	0.534
		BOLD	0.10	1.01	–0.11	1.00	0.253	0.998	0.456		
ROI 2	SPL	Thickness	0.41	0.78	–0.43	1.04	2.880	0.000	4.569	0.007*	0.841
		BOLD	–0.02	0.91	0.02	1.11	0.253	0.998	0.052		
ROI 3	IPL	Thickness	0.51	0.74	–0.53	0.97	3.799	0.020	9.221	0.001*	0.698
		BOLD	–0.07	0.84	0.08	1.17	0.245	0.998	0.009*		
ROI 4	SPL	Thickness	0.22	0.97	–0.23	1.00	0.167	0.020	3.373	0.158	0.437
		BOLD	–0.08	0.84	0.09	1.17	0.779	0.998	0.089		
ROI 5	FuG	Thickness	0.50	0.81	–0.53	0.92	4.968	0.003	6.877	0.001*	0.874
		BOLD	0.03	0.90	–0.03	1.13	0.245	0.998	0.020*		
ROI 6	IOG	Thickness	0.07	1.13	–0.07	0.86	1.167	0.001	0.208	0.656	0.283
		BOLD	0.17	0.91	–0.19	1.09	0.399	0.998	0.651		
ROI 7	SM1	Thickness	0.32	0.82	–0.34	1.08	1.671	0.022	2.805	0.037*	0.829
		BOLD	–0.05	0.88	0.06	1.14	0.365	0.998	0.111		
ROI 8	ITG	Thickness	0.35	0.79	–0.37	1.08	0.804	0.030	5.941	0.022*	0.278
		BOLD	–0.11	0.85	0.12	1.16	0.470	0.998	0.027*		
ROI 9	SM1	Thickness	0.32	0.76	–0.34	1.13	1.381	0.013	3.224	0.037*	0.718
		BOLD	–0.04	0.91	0.08	1.13	0.399	0.998	0.092		
ROI 10	MOG	Thickness	0.38	0.78	–0.40	1.06	2.794	0.012	3.594	0.012*	0.872
		BOLD	–0.04	0.90	0.04	1.13	0.253	0.998	0.082		
ROI 11	aIPS	T Thickness	0.54	0.64	–0.56	1.02	7.301	0.004	6.054	<0.001*	0.794
		BOLD	0.04	1.09	–0.04	0.95	0.245	0.998	0.027*		
ROI 12	IFG (vPMC)	Thickness	0.53	0.74	–0.55	0.95	4.030	0.016	9.703	<0.001*	0.540
		BOLD	–0.10	0.78	0.11	1.21	0.245	0.998	0.008*		
ROI 13	Precentral Gyrus (dPMC)	Thickness	0.65	0.58	–0.69	0.89	2.467	0.006	28.819	<0.001*	0.024*
		BOLD	–0.32	0.88	0.36	1.03	0.260	0.998	<0.001*		
ROI 14	MFG (vlPFC)	Thickness	0.46	0.78	–0.49	0.99	1.189	0.037	13.230	0.002*	0.167
		BOLD	–0.23	0.91	0.25	1.06	0.399	0.998	0.003*		
ROI 15	MFG (dPMC)	Thickness	0.48	0.71	–0.50	1.03	3.816	0.030	7.152	0.001*	0.825
		BOLD	–0.05	1.16	0.06	0.82	0.245	0.998	0.019*		
ROI 16	MFG (vlPFC)	Thickness	0.54	0.82	–0.57	0.85	3.977	0.006	12.168	<0.001*	0.606
		BOLD	–0.08	1.09	0.08	0.92	0.245	0.998	0.004*		
ROI 17	IFG (vPMC)	Thickness	0.46	0.94	–0.49	0.83	0.011	0.020	25.073	0.002*	0.001*
		BOLD	–0.46	0.51	0.51	1.17	0.955	0.998	<0.001*		
ROI 18	MFG (dPMC)	Thickness	0.47	0.86	–0.45	0.93	1.796	0.000	7.948	0.004*	0.356
		BOLD	–0.15	0.95	0.17	1.05	0.189	0.998	0.014*		
ROI 19	vlPFC/IFG	Thickness	0.40	0.85	–0.40	0.99	0.000	0.100	12.849	0.013*	0.016*
		BOLD	–0.37	0.58	0.41	1.21	0.998	0.998	0.003*		
ROI 20	IFG (vlPFC)	Thickness	0.61	0.70	–0.61	0.89	0.482	0.051	36.452	<0.001*	0.007*
		BOLD	–0.38	0.58	0.42	1.20	0.586	0.998	<0.001*		
ROI 21	IFG (vPMC)	Thickness	0.47	0.90	–0.47	0.89	0.090	0.007	17.186	0.003*	0.012*
		BOLD	–0.38	0.54	0.42	1.22	0.833	0.998	<0.001		
ROI 22	IFG pars orbitalis	Thickness	0.62	0.68	–0.66	0.86	2.675	0.000	31.249	<0.001*	0.109
		BOLD	–0.22	0.63	0.24	1.27	0.253	0.998	<0.001		
ROI 23	IFG	Thickness	0.46	0.77	–0.48	1.00	1.207	0.007	10.979	0.002*	0.162
		BOLD	–0.21	0.72	0.23	1.21	0.399	0.998	0.005*		
ROI 24	MFG (preSMA)	Thickness	0.50	0.72	–0.55	0.99	1.020	0.009	17.639	0.001*	0.060
		BOLD	–0.32	0.63	0.35	1.21	0.420	0.998	<0.001*		
ROI 25	MeFG	Thickness	0.47	0.71	–0.50	1.03	2.930	0.001	8.382	0.001*	0.595
		BOLD	–0.07	1.10	0.08	0.90	0.253	0.998	0.012*		

BA, Brodmann area; aIPS, Anterior Intra-parietal Sulcus; SPL, Superior Parietal Lobule; IPL Inferior Parietal Lobule; FuG, Fusiform Gyrus; SM1, Primary somatosensory cortex; ITG, Inferior temporal gyrus; Middle occipital gyrus; vPMC, Ventral premotor cortex; IFG, Inferior Frontal Gyrus; dPMC, dorsal premotor cortex; MFG, middle frontal gyrus; vlPFC, ventrolateral prefrontal cortex; oPFC, orbital prefrontal cortex; MeFC, medial frontal cortex.

*Statistically significant results.

### Between Group Comparison of Functional and Structural Data

A mixed factorial ANOVA to compare Group (Young, Elderly) and Index (BOLD-ROIs z-scores, Thick-ROIs z-scores) was performed (see Table 3). Results of ANOVA analysis are also reported in Table 3 and Figure 3. To summarize, no significant main effects of Group and Index were found. All significant Group × Index interactions were explored with *post hoc* analyses. Accordingly, significant Group x Index interaction with significant difference in cortical thickness in *post hoc* comparison was found in the left inferior parietal lobule (ROI 3, IPL, *p*_*FDR*__–__*corr*_ = 0.009), left fusiform gyrus (ROI 5, FuG, *p*_*FDR*__–__*corr*_ = 0.020), right inferior temporal gyrus (ROI 8, ITG, *p*_*FDR*__–__*corr*_ = 0.027), right anterior intraparietal sulcus (ROI 11, aIPS, *p*_*FDR*__–__*corr*_ = 0.027), left ventral premotor cortex (ROI 12, vPMC, *p*_*FDR*__–__*corr*_ = 0.008), left ventrolateral prefrontal cortex (ROI 14, vlPFC, *p* = 0.003), left dorsal premotor cortex (ROI 15, dPMC, *p*_*FDR*__–__*corr*_ = 0.019), left vlPFC (ROI 16, *p*_*FDR*__–__*corr*_ = 0.004), right dPMC (ROI 18, *p*_*FDR*__–__*corr*_ = 0.014), left pars orbitalis (ROI 22, IFG, *p*_*FDR*__–__*corr*_ < 0.001), left inferior frontal gyrus (ROI 23, IFG, *p*_*FDR*__–__*corr*_ = 0.005), right medial frontal cortex (ROI 24, preSMA, *p*_*FDR*__–__*corr*_ < 0.001), left MeFC (ROI 25, *p*_*FDR*__–__*corr*_ = 0.012). A significant Group x Index interaction with significant difference in both cortical thickness and BOLD signal in *post hoc* comparison was found in left dPMC (ROI 13, p < 0.001_*F*__*DR*__–__*corr*_), right vPMC (ROI 17, *p*_*FDR*__–__*corr*_ < 0.001), right vlPFC (ROI 19, *p*_*FDR*__–__*corr*_ = 0.003), right vlPFC (ROI 20, *p*_*FDR*__–__*corr*_ < 0.001), and right vPMC (ROI 21, *p*_*FDR*__–__*corr*_ < 0.001).

### Laterality Index

In the elderly group, the LI in the frontal lobe was –0.262 with a total voxel count 1333 on the left and 2281 on the right, and 0.239 for the parietal lobe with 3248 voxels on the left and 1994 on the right. In the young group, the LI in the frontal lobe was 0.586 with 1488 on the left and 389 on the right, and in the parietal was 0.637 with 5068 on the left and 1123 on the right.

## Discussion

Our aim was to explore the functional and structural modifications occurring in the MNS during normal aging. In particular, we found evidence of reduced cortical thickness and increased activation of the premotor cortices bilaterally, areas belonging to the MNS, and an additional involvement of right prefrontal cortices. Finally, unlike the young participants, the elderly group showed evidence of right frontal lateralization.

The whole sample showed an activation within a fronto-parietal network which comprised bilateral parietal areas (aIPS, IPL, SPL, primary sensorimotor cortex), vPMC and dPMC (middle and inferior frontal gyri, BA6, 44 and 45), classically considered MNS areas, and visual areas (BA37, 19), which do not belong to the MNS properly. Finally, right vlPFC (BA44-45) was also found.

The herein data agree with an extensive quantitative meta-analysis (Molenberghs et al., 2012) of fMRI data from 125 studies reporting the localization of the human MNS. The recruitment of the vPMC in the present study is in line with previous studies showing how the ventral sector of the premotor cortex is the most likely human equivalent of macaque mirror area F5 and the brain area where hand actions are represented (Binkofski et al., 1999; Rizzolatti et al., 2001; Rizzolatti and Craighero, 2004; Rizzolatti, 2005). Moreover, we also found the recruitment of dPMC, an area involved in the motor preparation of actions and in the observation of hand movements in association with the IFG (pars opercularis) (Buccino et al., 2001).

In our sample, we observed the recruitment of bilateral parietal areas centered in the IPS. Posterior parietal cortices are involved in the multimodal integration of information to construct a spatial representation of the external world. More specifically, the IPS can be considered a visuo-motor interface in the control of arm and eye movements in space for the object manipulation (Buccino et al., 2004; Grefkes and Fink, 2005; Vogt et al., 2007). Moreover, in agreement with previous reports (Gazzola et al., 2007; Cabinio et al., 2010), the MNS network included activation of the postcentral gyrus. This is in line with the role of the MNS in the mechanism of the internal simulation of the observed action. Thus, when we observe an action, a sensory motor resonance mechanism creates an internal subliminal reproduction of the sensory and kinematic aspects of the observed action.

Outside the strictly-defined MNS areas, we observed recruitment of temporal visual areas, corresponding to extrastriate body area (EBA) as previously described (Cabinio et al., 2010; Molenberghs et al., 2012) a region involved in the visual recognition of the human body (Downing et al., 2001; Urgesi et al., 2004).

The herein presented results confirmed the starting hypothesis of the presence of structural and functional modifications in MNS with normal aging, following the constraints of the age-related brain changes. Specifically, the frontal areas on both the right and left hemispheres were significantly different between the two groups for both fMRI activation and cortical thickness with the left dPMC, the right vPMC and right ventrolateral PFC showing increased activity in the elderly group coupled with a right frontal lateralization and reduced cortical thickness in the same areas. The dorsal and ventral PMC are part of the MNS (Rizzolatti and Craighero, 2004; Molenberghs et al., 2012), with a possible role in facilitating the motor output from the primary motor cortex through the motor resonance mechanism. Increased activation of these areas in the elderlies might suggest the necessity of increased activity by these areas to modulate and affect motor output from the primary motor cortex. Note that this implies that the PMC, as sector of the MNS, maintains its function to observe action, recognize them, and eventually allowing the individual to interact with one another.

Specifically, the observed PFC activation overlaps with the prefrontal area previously observed during a “learning-by-imitation” fMRI experiment in which musically naïve young subjects were asked to observe and then imitate the hand movement of a guitar chord (Buccino et al., 2004; Vogt et al., 2007). Buccino et al. (2004) interpreted the role of this PFC area in the selection of the appropriate motor act for the execution of the new motor acts (playing the guitar chord), to operate a recombination of the “resonated” motor acts into a new motor sequence. This finding is particularly interesting since participants in the present study the were asked to observe and execute very simple and well-known grasping actions.

The increased ventral PFC activation in the elderly group suggests a higher cognitive/attentive load probably related to a step-by-step planning of the motor action also during simple actions. One might argue that while in young healthy participants these prefrontal areas become active during the acquisition of novel motor tasks (e.g., when naïve participants learn to play some guitar chords following a model; Buccino et al., 2004; Vogt et al., 2007), in healthy elderly participants the recruitment of these areas always occurs also during the observation and recognition of actions that are already part of the participants’ motor repertoire. This in turn suggests that, as compared to younger people, the activation of these areas in elderly participants is necessarily a pre-requisite to process even for familiar and well-known actions. In other words, physiological aging is associated with the necessity to recombine simple motor acts *de novo* each time.

From a more cognitive point of view, the PFC is also widely associated with attention and executive-control systems and has been implicated in the allocation of resources (Badre, 2008; Badre and Nee, 2018). This area is typically recruited during cognitive tasks requiring attentional control and manipulation of information in working memory (Goldman-Rakic, 1995; Badre, 2008; Badre and Nee, 2018). Many neuroimaging studies investigating executive control have reported greater frontal cortex activation in older adults compared to younger adults (Nielson et al., 2002; Cabeza et al., 2004; Colcombe et al., 2005; Townsend et al., 2006; Kurth et al., 2016), supporting the hypothesis that this area may be linked to compensatory mechanisms in aging related to cognition (Cabeza et al., 2002). Taken together, these data are also relevant when planning rehabilitative intervention targeted to elderly individuals. Moreover, preliminary evidence showed how an action observation rehabilitation treatment was associated with improvements in attention and facial recognition in nursing home residents with dementia (Eggermont et al., 2009). Increased activation of premotor cortices with aging has been previously documented in several experiments investigating the motor system (Calautti et al., 2001; Hutchinson et al., 2002; Mattay et al., 2002; Heuninckx et al., 2005; Ward, 2006; Seidler et al., 2010; Wang et al., 2019; Tscherpel et al., 2020). The increased activation in the pre motor cortex can be interpreted as a compensatory mechanism specifically related to the MNS due to its well-established involvement in the MNS. However, considering the specificity of the mirror-task used, it is not possible to rule out a more general compensatory role in aging for this area.

In our study, the increased activation of the bilateral PMC and of the right PFC mentioned was coupled with reduced cortical thickness, a marker of neuronal loss and reduced brain reserve typical of the aging processes (Salat et al., 2004; van Velsen et al., 2013; Cabeza et al., 2018).

Our data are coherent with functional neurocompensatory models in aging such as the HAROLD model (Hemispheric Asymmetry Reduction in Older Adults) (Cabeza, 2002) that supports the reduction of lateralization of brain activity in aging, and the CRUNCH model (Compensation-Related Utilization of Neural Hypothesis Circuits) (Reuter-Lorenz and Cappell, 2008) that captures other aging related mechanisms consisting in the recruitment of additional brain regions to play out compensatory strategies to cope with the reduced brain reserve.

To the best of our knowledge, only one previous study explored aging and the MNS (Nedelko et al., 2010), comparing young versus elderly participants. In that study the seminal areas of the MNS did not show changes between groups. However, methodological differences might explain the discrepant results. Our paradigm, an execution-observation conjunction experimental design, allowed us to selectively define mirror areas, while Nedelko’s paradigm (Nedelko et al., 2010) was focused on action observation and imagery. The latter recruits a network only partially overlapping with the MNS (Gerardin et al., 2000).

The strength of our findings consists in this specific coupling of age-related measures obtained using two different MRI techniques (i.e., structural and functional MRI). The differential age effect observed in the brain reserve in frontal regions belonging to the MNS is associated both with an increased activity in the designated MNS (premotor) areas and an additional recruitment of non-specific MNS (prefrontal) areas. In light of this multimodal MRI study, we can hypothesize two possible mechanisms: compensation by up-regulation, if we consider the hyperactivity observed in the areas belonging to the MNS in the bilateral PMC, and compensation by reorganization if we consider the recruitment of right PFC observed in the elderly (Cabeza et al., 2018). More generally, according to the CRUNCH model, our data can be considered as evidence for an important role of this area as a brain reserve hub, thus involved in the aging process of several domains.

A final remark concerns the characteristics of the elderly participants included in the study. Results from the neuropsychological assessment showed that they performed within the normal range on all the neuropsychological tests, confirming that they were globally preserved in cognitive functioning and thus representative of the normal aging population.

Although the findings of the current investigation provided deeper understanding of the functional organization and structural brain reserve of the MNS in healthy older adults, there are some limitations that need to be considered. First, we selected participants that underwent a single session study, but we did not include longitudinal evaluations. A future study should provide further evidence with a longitudinal design and explore the age-related monitoring changes during normal aging. Second, the lack of reserve measures in these participants can limit the comprehension of aging process. Future studies investigating the correlation between neuroimaging data and aspects of reserve (such as cognitive reserve) will be necessary to account for inter-individual variability in aging.

## Conclusion

In conclusion, our data suggest that during aging, the MNS is subject to both structural and functional modifications resembling what occurs in the neuromotor and neurocognitive aging. At a structural level, the MNS undergoes cortical thinning; whereas from a functional level, its activation increases bilaterally in the premotor cortices with additional recruitment of right prefrontal cortex.

## Data Availability Statement

The raw data supporting the conclusions of this article will be made available by the authors, without undue reservation.

## Ethics Statement

The studies involving human participants were reviewed and approved by Ethics Committee section of “IRCCS Fondazione Don Carlo Gnocchi,” part of the IRCCS Ethics Committee of Regione Lombardia. The patients/participants provided their written informed consent to participate in this study.

## Author Contributions

SD, VB, MC, GB, and FB contributed to conception and design of the study. SD, VB, NB, and MC performed the statistical analysis and wrote the first draft of the manuscript. SD, VB, MC, NB, and GB contributed to manuscript revision, read, and approved the submitted version. All authors contributed to the article and approved the submitted version.

## Conflict of Interest

The authors declare that the research was conducted in the absence of any commercial or financial relationships that could be construed as a potential conflict of interest.

## Publisher’s Note

All claims expressed in this article are solely those of the authors and do not necessarily represent those of their affiliated organizations, or those of the publisher, the editors and the reviewers. Any product that may be evaluated in this article, or claim that may be made by its manufacturer, is not guaranteed or endorsed by the publisher.

## Footnotes

1
http://www.pstnet.com

2
http://www.fil.ion.ucl.ac.uk/spm

3
http://cibsr.stanford.edu/tools/human-brain-project/artrepair-software.html

4
http://marsbar.sourceforge.net

5
http://imaging.mrccbu.cam.ac.uk/downloads/MNI2tal/mni2tal-m

6
http://www.freesurfer.net/

7
www.fil.ion.ucl.ac.uk/spm/ext/
